# Multisensory Fusion for Unsupervised Spatiotemporal Speaker Diarization

**DOI:** 10.3390/s24134229

**Published:** 2024-06-29

**Authors:** Paris Xylogiannis, Nikolaos Vryzas, Lazaros Vrysis, Charalampos Dimoulas

**Affiliations:** Multidisciplinary Media & Mediated Communication Research Group (M3C), Aristotle University, 54636 Thessaloniki, Greece; pxylog@jour.auth.gr (P.X.); nvryzas@jour.auth.gr (N.V.); lvrysis@auth.gr (L.V.)

**Keywords:** speaker diarization, sound localization, AI-enabled systems, multimodal decision making, deep learning, smartphones

## Abstract

Speaker diarization consists of answering the question of “who spoke when” in audio recordings. In meeting scenarios, the task of labeling audio with the corresponding speaker identities can be further assisted by the exploitation of spatial features. This work proposes a framework designed to assess the effectiveness of combining speaker embeddings with Time Difference of Arrival (TDOA) values from available microphone sensor arrays in meetings. We extract speaker embeddings using two popular and robust pre-trained models, ECAPA-TDNN and X-vectors, and calculate the TDOA values via the Generalized Cross-Correlation (GCC) method with Phase Transform (PHAT) weighting. Although ECAPA-TDNN outperforms the Xvectors model, we utilize both speaker embedding models to explore the potential of employing a computationally lighter model when spatial information is exploited. Various techniques for combining the spatial–temporal information are examined in order to determine the best clustering method. The proposed framework is evaluated on two multichannel datasets: the AVLab Speaker Localization dataset and a multichannel dataset (SpeaD-M3C) enriched in the context of the present work with supplementary information from smartphone recordings. Our results strongly indicate that the integration of spatial information can significantly improve the performance of state-of-the-art deep learning diarization models, presenting a 2–3% reduction in DER compared to the baseline approach on the evaluated datasets.

## 1. Introduction

Speaker diarization is an essential task in many applications, addressing the modern needs of human–computer interaction and semantic segmentation in the directions of content management and knowledge extraction. While it has seen great improvements as a field in the past decade, it is still a difficult task, and its state-of-the-art performance is far from perfect. Several limitations, including the time resolution of speaker diarization systems, the presence of overlapping speaker segments, and noise in audio recordings, can undermine the performance of modern diarization systems. Additionally, the complexity of real-world audio, with background conversations, varying accents, and emotional inflections, compounds these challenges and introduces further restrictive factors for achieving robust accuracy.

Traditional systems rely on individually optimizing multiple independent submodules. Such systems typically first involve stages of audio preprocessing, speech activity detection, and segmentation. Subsequently, most diarization approaches derive from the well-established idea of extracting an audio features vector from each segment of a speech signal and using it as an identity vector to be fed into a clustering module. Early systems used metric-based algorithms such as the Bayesian information criterion (BIC) [1,2] for the similarity measurement between speech segments. Later, the Gaussian Mixture Model-based Universal Background Model (GMM-UBM) [3] became the standard for speaker verification tasks and Joint Factor Analysis (JFA) [4,5] was then proposed as an effective improvement using low-dimensional speaker representations named i-vectors. With the emergence of deep learning, several neural speaker representation approaches were proposed [6,7,8], mainly based on the bottleneck layer output of a deep neural network (DNN) trained for speaker recognition and verification tasks. Compared to traditional factor analysis methods, DNN-based speaker embeddings offer a greater accuracy and computational efficiency during inference. Recent advances in deep learning have greatly impacted the speaker diarization field with approaches based on graph neural networks (GNN) [9,10] and transformer architectures [11,12]. In modern automatic speech recognition (ASR), speaker diarization is a crucial processing step [13] of the system, highlighting the need to further improve the performance of speaker labeling tasks.

In [14], the authors take advantage of the similarity of the tasks of speech separation and speech diarization to implement a speech-separation-guided diarization (SSGD) approach, achieving a DER of 12.81–14.28% in a dataset with two speakers. A lower DER matches bigger training data. In [15], a speech-separation-guided system along with a dual-path RNN model is proposed, achieving a DER of 11.1% in phone call speaker diarization. Memory-aware speaker embedding mechanisms are introduced in [16], leading to a DER of 11.12–14.04% for the best-performing configuration. A sequence-to-sequence architecture that incorporates memory-aware multi-speaker embedding is used in [17], resulting in a DER of 15.9% on the CHiME-7 evaluation dataset. A Dynamic Bayesian Network (DNB) is proposed in [18] for audiovisual diarization, achieving a DER of 15.32% on the AVDIAR dataset. An End-to-End Neural Speaker Diarization (EENSD) method with an attention mechanism is evaluated in [19], achieving a DER of 10.99% on the Callhome dataset. In [20], an EENSD architecture leads to a DER of 12.28%, which is a great improvement compared to the baseline clustering method that gives a DER of 28.77%.

Multimodal systems for speaker diarization that incorporate information from multiple sources have been proposed. Audiovisual systems are the most common type of multimodal diarization, combining audio and video data [21,22]. By exploiting visual cues such as facial expressions and lip movements, these systems can achieve an improved accuracy, especially in challenging environments with background noise or overlapping speech. In [23], a multimodal audiovisual framework to provide Web-TV automations for live broadcasting integrating speaker diarization is proposed. The development of multimodal systems is still an active area of research. Challenges include the synchronization of audio and video streams, the extraction of meaningful visual features, and the effective fusion of these distinct modalities.

Another important subfield of multimodal diarization includes spatiotemporal systems. Speaker diarization systems that exploit the spatial characteristics of speech and sound have been proposed in previous related studies. An earlier approach focused on the time delay between the pairs of microphones to construct a similarity matrix of the sound segments [24]. Other works [25] benefit from acoustic beamforming techniques to construct single-channel reference audio that enhances spectral information extraction. Additionally, MFCC and TDOA values can be combined to form a spatiotemporal information domain [26]. Another spatial approach for speaker diarization utilizes Direction of Arrival (DOA) information [27]. In a related study [28], Multiple Distant Microphones (MDMs) were employed to extract DOA values and combine them with acoustic feature information. A recent study [29] utilizes spatial information to implement a real-time speaker diarization system. Another work proposes a neural network architecture with multichannel input [30]. Our previous work [31] focused on information fusion between speaker embeddings from DNN and TDOA values from microphone pairs.

The motivation for the current work lies in designing a generic framework that incorporates different types of heterogeneous devices in arbitrary setups. The information from audio signals from multiple types of sensors can be used in an information fusion scheme to address the complex task of speaker diarization. AI-enabled smart systems for speaker diarization have useful applications for several solutions and workflows for content production, communication, and decision making. While many of the technologies involved are mature enough to be used in production, the research hypothesis is that there is still important added value that can be gained by a combination of different modalities. Moreover, the use of different setups is promoted without the need for additional user effort for the calibration and synchronization of the sensors or investment in dedicated equipment.

The proposed system in this particular work combines information from the two domains to enrich speaker representations that are used as inputs in unsupervised clustering algorithms. This study aims to extend and improve this method, examining different information combinations and clustering approaches. More specifically, various experiments are conducted to explore richer and more robust speaker representations that efficiently incorporate a balanced mixture of information from both microphone arrays and acoustic speech features.

The remainder of the paper is structured as follows: in Section 2, the datasets that were used are described, as well as the different modules involved and their fusion, in Section 3, the experimental results are presented and discussed, and in Section 4, the research is concluded and the future work plans are stated.

## 2. Materials and Methods

### 2.1. Datasets

**Figure 1:**
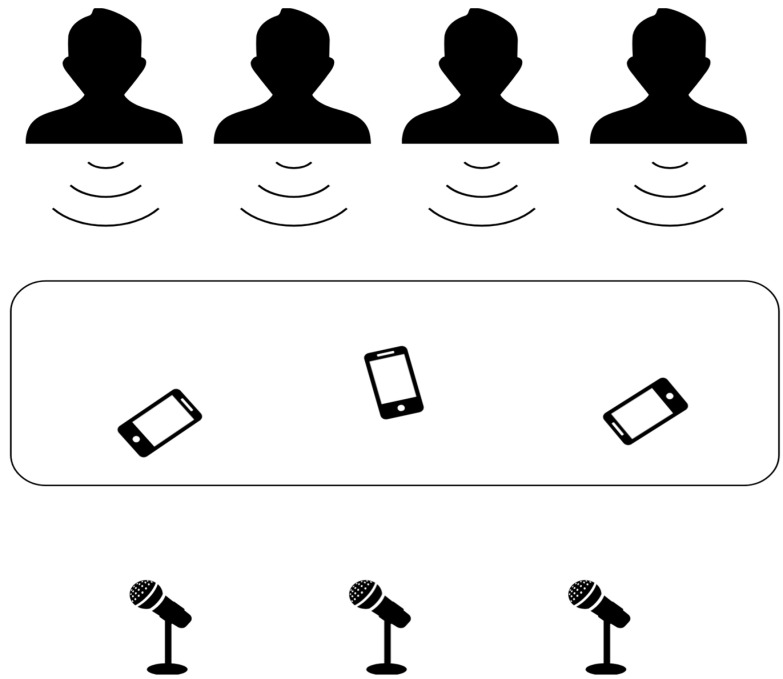
A visual representation of the Spead-M3C dataset formulation layout, featuring four speakers and three aligned microphones with the addition of three randomly positioned smartphones.

To thoroughly evaluate the performance of our system in various microphone pair configurations, we strategically exploited two datasets that were rigorously created for multichannel speech-related tasks. Our initial experimentation was centered around the AVLAB speaker localization dataset [32]. The AVLAB dataset simulates a dynamic talk-show panel environment, with three to four speakers engaged in conversation. Its dual-microphone array, arranged in a classical A-B arrangement, facilitates the accurate calculation of a single TDOA (Time Difference of Arrival) value for each time segment within a recording. Experiments utilizing this particular dataset can provide a robust basis for understanding the behavior of the system under well-controlled conditions. Pursuing testing our system with more complex scenarios, we incorporated the SpeaD-M3C dataset. Designed to facilitate our previous research, SpeaD-M3C retains the fundamental speaker and sensor layout of the AVLAB dataset. However, it is decisively distinguished using three aligned microphones. This expanded layout allowed us to explore the performance of our system with a larger number of microphone pairs, providing deeper insights into its diarization capabilities. To further extend our evaluation and introduce an element of real-world variability, we extended our tests to include audio conversations recorded from three smartphones. These devices were randomly placed in front of the speakers, simulating the less predictable acoustic environments often found in practical applications. This unique testing approach allowed us to measure the robustness and efficiency of our system when faced with the possibility of non-synchronized microphone sensors with varying specifications and a less-than-ideal microphone placement. This multifaceted approach allowed us to identify strengths, highlight potential areas for improvement, and ultimately deliver a speaker diarization system optimized for deployment in various real-world environments. The Spead-M3C speaker and microphone sensor setup is demonstrated in Figure 1. Both datasets can be accessed at https://m3c.web.auth.gr/research/datasets/ (accesed on 27 June 2024).

### 2.2. Speaker Embeddings for Diarization

Two successful neural network architectures that stand out as highly effective methods for extracting speaker representations were used in the present work: X-vectors [33] and ECAPA-TDNN [34].

The X-vectors model architecture consists of various crucial parts. Initially, the network receives audio inputs such as Mel-Frequency Cepstral Coefficients (MFCCs) or Filterbank energies. These input frames are processed by a number of TDNN layers at different levels. The TDNN layers are deliberately constructed to extract short temporal patterns from the acoustic features, and they are, therefore, well-suited to speech analysis. One key aspect of the X-vector architecture is the statistics pooling layer. This particular type of neural network layer has the ability to handle utterances that differ in length among segments. More specifically, the average statistics pooling layer combines all the representations generated by the TDNN layers over an entire utterance into a single fixed-length vector through calculating first-order (mean) and second-order (centered variance) statistics. Finally, segment-level layers analyze the pooled information further. These fully connected layers target high-level, long-term properties of speaker’s speech occurring across each full utterance. The inputs to the network are 24-dimensional log Mel Filterbank energies and the generated output is a 512-dimensional speaker embedding vector. The model’s architecture, as originally described in [33], is shown in Table 1. As presented, the model contains 4.2 million parameters.

**Figure 2:**
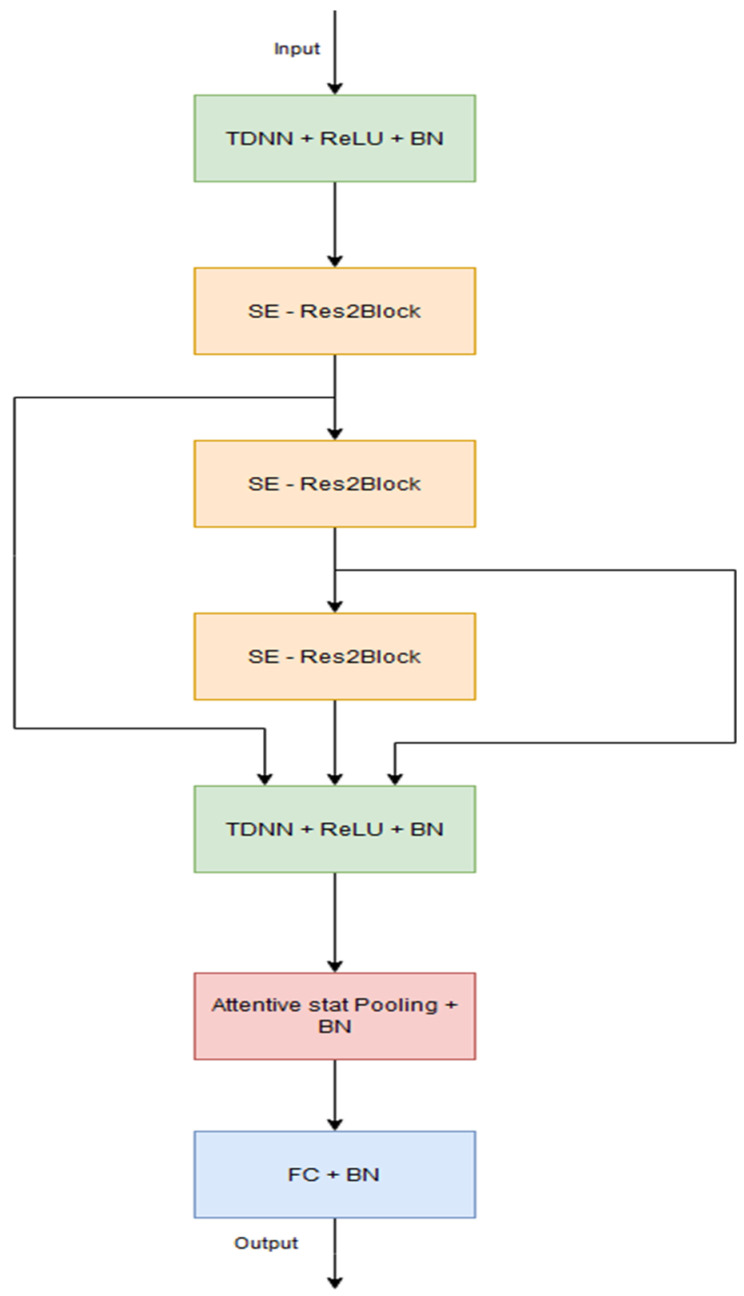
The ECAPA-TDNN model architecture.

ECAPA-TDNN evolves the X-vectors’ framework with essential modifications. The time delay convolutional layers are replaced by complex one-dimensional Res2Net units embedded with skip connections. This enables the network to distinguish progressively nuanced patterns. In addition, Squeeze-and-Excitation (SE) blocks are integrated into these modules, intelligently upscaling the feature responses across channels to highlight those most critical for speaker diarization. Finally, ECAPA-TDNN augments the statistics aggregation module with the integration of attention mechanisms, allowing the network to zero in on highly relevant input regions for even more robust speaker representation. The model uses 80-dimensional log Mel Filterbank energies as inputs and generates 192-dimensional representations. The architecture is presented in Figure 2. In the presented study, we employ the variation using 1024 filters *C* in the convolutional layers. Both TDNN layers have a dilation spacing of 1 and kernel sizes of 5 and 1, respectively. All SE—Res2Blocks use a kernel size of 3 with increasing dilations starting from 2 at the first block. This specific model variation has 14.7 million parameters.

It is apparent that these architectures have different computational requirements. The use of two models originates from our interest to investigate the potential benefits of adding spatial speech information to the produced representations. Comparing the performances of these models will provide valuable insights into the trade-off between the computational complexity and robustness of speaker embeddings when incorporating spatial information derived from microphone arrays.

### 2.3. Time Delay of Arrival Information Extraction

The proposed system focuses on speaker diarization in the context of meeting scenarios, where speakers often maintain relatively stationary positions. A typical example of such a real-world scenario is the recording of a meeting using smartphones placed randomly on a table in front of the participants. In this setting, the primary objective of providing the necessary meeting information is less about accurately identifying the speakers’ locations and more about identifying who is speaking at a given time. Due to the unique dynamics of these scenarios, TDOA values emerge as the preferred spatial characteristics of the proposed pipeline. These TDOA values are calculated for each speech segment through the Generalized Cross-correlation with Phase Transform (GCC-PHAT) [35] applied to all microphone pairs. This method has several advantages. First, GCC-PHAT works reliably even when the number of microphones is varied and their exact locations within the conference room remain unknown. Second, for each audio segment, a unique TDOA vector can be generated that corresponds to the time delay for all microphones pairs within the sensor array. Although this arrangement does not facilitate accurate arrival directions, it yields valuable information about the unique, albeit unknown, location of each speaker. Finally, the length of the TDOA vector for each speech segment directly corresponds to the number of available microphone pairs. Therefore, in cases where large arrays of microphones are available, the spatial information vector can greatly affect the speaker representations. As described in [36], given two signals, *x_i_*(*n*) and *x_j_*(*n*), the GCC-PHAT is defined as:

(1) $$G_{PHAT}\left(f\right)=\frac{X_{i}\left(f\right)\bar{\;X_{j}\left(f\right)}}{\left|X_{i}\left(f\right)\;X_{j}\left(f\right)\right|}=e^{j\left(\varphi_{i}\left(f\right)-\varphi_{j}\left(f\right)\right)\;},$$

where *X_i_*(*f*) and *X_j_*(*f*) are the Fourier transforms of the two signals and (¯) denotes the complex conjugate. The last part of Equation (1) encapsulates the phase difference between the two signals in the frequency domain, where *φ_i_*(*f*) and *φ_j_*(*f*) are the corresponding phase functions of these signals. If we denote the inverse Fourier transform of Equation (1) as *R_phat_*(*d*), then the TDOA for this pair of signals is estimated as:

(2) $$d_{PHAT}\left(i,\;j\right)={argmax}_{n}R_{PHAT}{\left(n\right)}_{\;},$$

### 2.4. Spatiotemporal Information Fusion

**Figure 3:**
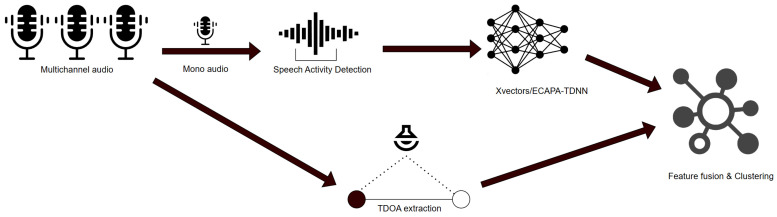
The proposed speaker diarization system with a joint spatiotemporal information pipeline.

Our proposed system is based on the foundations of traditional speaker diarization frameworks, as shown in Figure 3. It consists of several interconnected submodules, each of which contributes significantly to the overall performance. Initially, the mean of the audio channels is fed as a single-channel input to a Speech Activity Detection (SAD) model in order to distinguish speech segments from non-speech content. For our experiments, we used the pre-trained Silero VAD model [37] to obtain speech segments. This particular sub-module is flexible and can be adapted with alternative SAD implementations. We split the detected speech segments into overlapping time windows of equal lengths to facilitate subsequent analysis. Two main processes operate simultaneously in these time windows. First, the GCC-PHAT algorithm is used to process the multichannel audio input and compute the TDOA values between pairs of microphones. Second, neural network models are used to generate speaker embeddings with the single audio channel as an input.

The features extracted from both main modules must be combined in order to examine the impact of spatial features in the diarization task and explore the presence of complementary information between those and speaker embeddings. In our previous work, we simply concatenated both feature types to form a single speaker representation vector. The combined feature vector was then fed as an input to a Spectral Clustering (SC) algorithm [38] to extract the speaker labels for each time window. In all our experiments involving spectral clustering, we employed the auto-tuning implementation [39] to automatically estimate the number of speakers in a recording, with pruning applied to refine the affinity matrix and only keep the 10% values with the strongest similarity. Although this approach yields better results than simply using only the DNN models, it is important to explore the influence of each feature vector. Particularly, the high dimensionality of speaker embeddings will naturally impose a greater impact on cluster generation. Consequently, the influence of TDOA values with a much lower dimensionality may be reduced when determining the overall distance relationships between speaker identities. Therefore, we propose two other methods to investigate the construction of richer speaker representations for more robust diarization.

Our first approach to mitigating any potential bias in the clustering process is based on calculating a weighted similarity matrix as an initial step toward spectral clustering. Instead of generating a single affinity matrix for all samples and features, we compute separate similarity matrices for speaker embeddings and TDOA values. The objective of this specific implementation is to study the relationship between the information within the spatial and temporal domains. The Weighted Similarity Matrix is calculated using both speaker embeddings and TDOA values defined as follows:

- *S_i_* and *S_j_* are the speaker embeddings for samples *i* and *j*, respectively. Each embedding is a vector of dimension d.
- *t_i_* and *t_j_* represent the TDOA vectors for the same samples, each of dimension m.

The similarity between speaker embeddings is calculated using cosine similarity:

(3) $$Cosine\;Similarity=\frac{S_{i}\cdot S_{j}}{\left\|S_{i}\|\;\|S_{j}\right\|}\;,$$

where (·) denotes the dot product and (‖ ‖) denotes the Euclidean norm.

The similarity based on TDOA is computed using the Euclidean distance, transformed into a similarity measure:

(4) $$TDOA\;Similarity=\frac{1}{1+\|t_{i}-t_{j}\|}\;,$$

where $\left(\left\|t_{i}-t_{j}\right\|\right)$ is the Euclidean distance between the TDOA vectors $\left(t_{i}\right)$ and $\left(t_{j}\right)$.

Given that there are *n* samples, the Weighted Similarity Matrix, $\left(W\right)$, is an $\left(n\times n\right)$ matrix, where each element $\left(W_{ij}\right)$ is computed as:

(5) $$W_{ij}=w\times \left(\frac{S_{i}\cdot S_{j}}{\left\|S_{i}\|\;\|S_{j}\right\|}\right)+\left(1-w\right)\times \left(\frac{1}{1+\|t_{i}-t_{j}\|}\right)\;,$$

where $\left(w\right)$ is a weighting factor between 0 and 1 that balances the influence of speaker embeddings and TDOA vectors in the similarity calculation.

In our experiments, we additionally present a variation with cosine similarity for both parts of the equation, although the method of exploiting the Euclidean distance to calculate the similarity demonstrates better results in most cases. The fused similarity matrix, containing a joint representation of all feature types for each segment, becomes the input for the subsequent steps of spectral clustering. In order to investigate the optimal information combination between these domains, we run experiments with multiple weight values during the similarity matrix computation process.

Τhe last clustering approach in our experiments introduces a hierarchical element to the speaker cluster estimation. In the first stage, spectral clustering is performed only on the speaker embeddings for all samples. This initial step groups speech segments based on their acoustic feature similarity. As TDOA vectors’ dimensionality heavily depends on microphone pairs’ availability and is, therefore, much lower than embedding features, we explore the potential of treating the initial clusters produced by spectral clustering as numerical features to ensure an equal influence from both spatial and temporal domains on the final clustering. The encoded cluster features are then concatenated with the TDOA values for each speech segment. Finally, we employ the K-means clustering algorithm using the concatenated representation as an input. We set the number of clusters for the K-means to correspond to the number of clusters estimated by the auto-tuning spectral clustering algorithm. In our experiments, we examine if this method can potentially correct mislabeled time windows that occur due to speech acoustic similarity between speakers in different positions.

### 2.5. Synchronization of Smartphone Recordings

A major part of our study consists of studying the system’s performance using audio recordings from asynchronized sensors. This particular scenario is found in abundance in real-life situations where speaker diarization is necessary. For instance, in meeting scenarios, every participant can set a personal smartphone recording audio in a random position and then use the spatial information created to extract valuable meeting data and transcriptions. To extract meaningful spatial features, it is necessary to synchronize the audio channels. More specifically, in the non-synchronized smartphone recordings part of our dataset, every microphone has different start and stop timestamps. Although it is possible to directly calculate the TDOA values, we perform an optional preprocessing step to mitigate potential undermining factors, such as noise and other audio events introduced in different recording parts that not all channels have captured. To address this issue, we employ a simple but effective synchronization method. First, we segment the signals in small overlapping windows and calculate the RMS envelope for each segment. We then perform cross-correlation on the RMS envelopes of corresponding channels to identify the initial time shift. One audio channel is selected as a reference in order to synchronize the others accordingly. This practical approach significantly increases the system’s efficiency and, therefore, we include it as a preprocessing step for asynchronized multichannel datasets.

## 3. Results and Discussion

### 3.1. Optimal Time Window Selection

In a speaker diarization system, the choice of an appropriate time window for feature extraction is crucial. The length of the available time segments directly affects the quality of the speaker embeddings and the temporal resolution of time delay calculations, introducing a trade-off between the two domains. Longer time windows provide a richer and denser source of data for DNN embeddings’ generation. By capturing more speech features, including variations in time, these speaker representations become more robust and discriminative. On the other hand, a larger window increases the possibility of including time segments with overlapping speakers or longer durations of noise and silence. Such inclusions may degrade the quality of the speaker embeddings and the subsequent diarization process. On the contrary, a smaller time window reduces the possibility of speaker overlap or unwanted noise, but potentially results in less informative speaker representations. In addition, TDOA estimations through GCC-PHAT benefit from analyzing smaller time windows, allowing for more accurate discriminative spatial information. Moreover, we based our choice of selected time windows on previous work [23,31,40] focusing on speaker diarization adapted to production settings. A wider window than 1.5 s is considered to be too wide for several applications on production automation, while a window narrower than 0.5 s produces suboptimal results.

To determine an optimal time window length for our system, we conducted experiments using three different overlapping window sizes: {1.5, 1, 0.5} seconds. We applied a 50% overlap to ensure smooth transitions and efficient signal processing calculations. Our initial experiments showed that a 1 s window with a 0.5 s overlap consistently provided the best performance across all metrics we evaluated. Therefore, we selected this specific time window for use in all following experiments.

### 3.2. Evaluation Metric

We evaluate the accuracy of our system using the diarization error rate [41]. The DER computes the combined rate of three distinct error categories: false alarm (FA), non-speech segments mislabeled as speech, the missed detection of speech segments, and speaker label confusion. The total duration of errors is divided by the total duration of the reference signal to compute the final result. It is defined as follows:

(6) $$DER=\frac{False\;Alarm+Missed\;Detection+Speaker\;Confusion}{Total\;Duration}\;,$$

### 3.3. Evaluation of Feature Concatenation Method

In Table 2, we present our system’s results employing the first approach described, in which speaker embeddings and TDOA values are directly concatenated. The results are the same as those provided in our previous study with the addition of the asynchronized smartphone recordings part of SpeaD-M3C. We denote this particular subset as A-Spead in the result tables.

**Figure 4:**
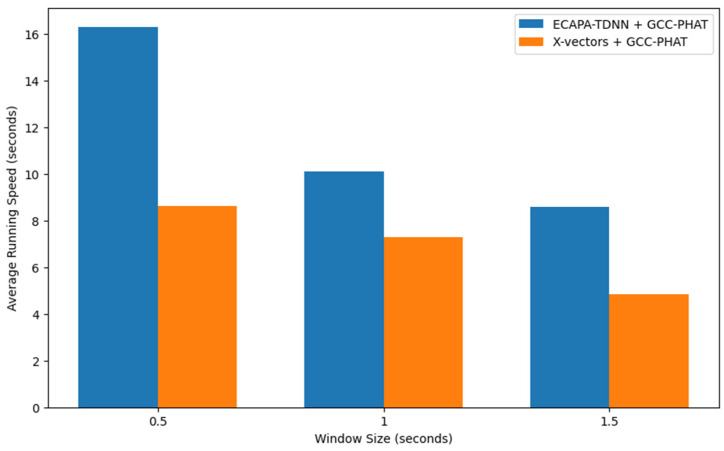
Comparison of average running speed by model and window size.

As demonstrated, our proposed system presents an improvement in the DER compared to when only using the DNN model, indicating the importance of incorporating spatial information into the speaker diarization process. Moreover, this reduction in DER is more pronounced with the dataset containing a larger number of microphone pairs. It is evident that the addition of more TDOA values enriches the construction of speaker representations, enhancing the ability of the system to accurately distinguish speakers. Furthermore, our proposed method offers an advantage in computational efficiency. In Figure 4, a comparison of the average running speed between the two models is presented. The best performing variation—the X-vectors architecture with the GCC-PHAT algorithm—turned out to be less computationally demanding than the ECAPA-TDNN model. In addition, it is essential to take into consideration that the X-vectors model is significantly more parameter-efficient, with only 4.2 million parameters compared to the 14.7 million parameters in the ECAPA-TDNN, as described in Section 2.

This result is important when considering real-world scenarios. as it suggests that we can achieve a lighter and more efficient solution for diarization without compromising accuracy.

### 3.4. Optimal Information Combination Exploration

As mentioned in the previous section. we build upon the aforementioned method to investigate an optimal spatiotemporal information combination. In our weighted similarity matrix approach, we intend to systematically examine the varying impacts of speaker embeddings and TDOA values information on the diarization process. In order to achieve this, we explore various weight values within the set w = {0, 0.25, 0.5, 0.75, 1}. This varying weight set will determine the relative contribution of neural network embeddings and TDOA features to the calculation of pairwise similarities between speech segments. In Table 3, we present the results for the two additional methods. The spectral clustering method employing a weighted matrix calculation is denoted as Similarity Matrix Fusion (SMF). We assign two different prefixes, C-SMF and E-SMF, depending on the use of cosine similarity or Euclidean similarity for the calculation of the spatial information affinity matrix. Our last approach, utilizing a final K-means algorithm after first clustering the speaker embeddings and computing a number of clusters estimation, is denoted as Hybrid Diarization Clustering (HDC).

As shown in the table, the similarity matrix fusion method demonstrates the most significant improvement in performance compared to simply concatenating the features in each sample. More specifically, when employing intermediate weights (w = 0.5 and w = 0.75), this method is far superior to other variations of the system. This finding suggests that the weighted similarity matrix clustering utilizes information from both speaker embeddings and TDOA more effectively when it assigns a balanced importance to each information domain. Furthermore, while in the concatenated features approach, the use of the X-vectors’ architecture resulted in the lowest DER, with this particular clustering algorithm, ECAPA-TDNN with GCC-PHAT outperforms other methods on all experiments. Overall, the superior performance of SMF with intermediate weight values reveals the potential of effectively exploiting spatial information in situations where the acoustic speech features and, therefore, the generated speaker embeddings are not adequate to correctly distinguish between similar speakers.

Finally, the evaluation of the hybrid diarization clustering method indicates that this method does not consistently outperform the performance achieved through feature concatenation. Although in most cases, HDC results in a slightly lower diarization error, the results are less optimal compared to weighted similarity matrix spectral clustering, as demonstrated in Table 3.

### 3.5. Limitations

The presented research aimed at addressing the problem of arbitrary microphone placement for the speaker diarization problem. For this reason, in all experiments, the microphones were placed randomly and information regarding their positions was not taken into account in the methodology. Several assumptions were made that introduce some limitations into our methodology. First, the microphone positions were fixed throughout the experiment, as well as their orientation. The experiment considered a maximum of four speakers, which is a common scenario in media production, but may not be applicable in other cases, like a conference. The experiment was conducted in a quiet and treated room, with minimal effect of noise, reverberation, etc. There was no overlapping of the speakers and no presence of non-speech elements (like coughing, laughter, etc.).

## 4. Conclusions and Future Work

In this study, we investigated the effectiveness of several methods for dealing with spatiotemporal information in the form of speaker embeddings generated from two different DNN architectures and the time difference of arrival values in a joint speaker diarization system. Through different system variations, we examined three primary clustering approaches: the spectral clustering of concatenated spatiotemporal features, similarity matrix fusion based on the calculation of distinct weighted affinity matrices for each information domain, and hybrid diarization clustering in which we implemented a sequence of clustering algorithms calculating first speaker embeddings clusters and then integrating spatial information for a final K-means clustering.

The initial evaluations showed that concatenating speaker embeddings with TDOA values yielded a better performance than only using speaker embeddings. The system resulted in a lower DER, highlighting the benefit of incorporating spatial information into speaker diarization. Further experimentation revealed that the weighted similarity matrix method consistently outperformed all other approaches, especially when assigning a balanced influence for embeddings and TDOA data. We speculate that the superior performance of SMF was due to its ability to facilitate a more refined integration of information from both feature domains. These evaluation results suggest that further exploration of the weight configurations and parameter optimization of the calculated affinity matrix can potentially lead to an even lower diarization error rate. Future studies could focus on developing adaptive algorithms that dynamically adjust the fusion parameters based on the variability of the input features, potentially improving the robustness and accuracy of the diarization process. Furthermore, although our two-stage diarization clustering method was outperformed by SMF, in most cases, it demonstrated better results than feature concatenation. This result indicates that it can be a fast and simple viable approach in scenarios where an exhaustive weight value calculation is not possible. In all cases, the proposed method resulted in very promising DER values, compared to the SOTA methods [14,15,16,17,18,19,20] that are evaluated in various multi-speaker datasets and are referenced in the Introduction. This strengthens the feasibility of the proposed architecture for addressing real-world problems. Due to the nature of the proposed methodology, which relies on the presence of multi-channel audio information, it is not possible to evaluate our system in a direct comparison with SOTA models. It is, however, within future plans to implement different combinations of models with the TDOA addition to evaluate the advantages of utilizing spatial information in speaker diarization.

In future work, it is planned to experiment with different arbitrary microphone and smartphone arrays, including video capturing, and implement and evaluate the system as an integrated solution for real-world applications. Evaluation with different arrangements, non-fixed microphone positionings, and orientations is also considered. The problems of speech overlapping and unwanted noise source inference should also be addressed. Finally, the inclusion of VAD as a module of an end-to-end system is valuable for deployment in real-world applications.

## Figures and Tables

**Table 1:** The X-vectors model architecture. T corresponds to the number of input frames.

Layer	Layer Contenxt	Total Context	Input × Output
frame1	{t – 2, t + 2}	5	120 × 512
frame2	{t – 2, t, t + 2}	9	1536 × 512
frame3	{t – 3, t, t + 3}	15	1536 × 512
frame4	{t}	15	512 × 512
frame5	{t}	15	512 × 1500
stats pooling	{0, T}	T	1500 × 3000
segment6	{0}	T	3000 × 512
Number of paramaters: 4.2 million			

**Table 2:** DER metric results of the proposed feature concatenation diarization system. Bold numbers indicate the best performing model.

	AVLAB	SpeaD-M3C	A-SpeaD
Xvectors	13.6%	11.6%	12.3%
Xvectors + GCC-PHAT	12.4%	9.2%	10.6%
ECAPA	13.2%	10.7%	11.5%
ECAPA + GCC-PHAT	12.6%	9.5%	10.8%

**Table 3:** Full presentation of DER results on all speaker diarization datasets. Bold numbers indicate the best performing model.

Dataset	System	E-SMF					C-SMF					HDC
		0	0.25	0.5	0.75	1	0	0.25	0.5	0.75	1	
AVLAB	ECAPA	14.1%	13.8%	12.4%	**11.8%**	13.2%	15.8%	14.6%	12.6%	**12%**	13.2%	12.2%
	Xvectors	14.1%	13.5%	**12.1%**	12.4%	13.6%	15.8%	14.4%	12.4%	**12.2%**	13.6%	12.4%
Spead-M3C	ECAPA	11.9%	11.2%	9.8%	**8.9%**	10.7%	12.3%	11.7%	9.6%	**9.2%**	10.7%	9.3%
	Xvectors	11.9%	11.4%	9.4%	**9.1%**	11.6%	12.3%	11.2%	**9.2%**	9.5%	11.6%	9.6%
A-Spead	ECAPA	12.6%	11.7%	10.6%	**9.8%**	11.5%	13.2%	12%	10.5%	**10.1%**	11.5%	10.4%
	Xvectors	12.6%	11.9%	10.9%	**10.5%**	12.3%	13.2%	12.3%	**10.6%**	10.9%	12.3%	10.5%

## Data Availability

The data are available upon reasonable request from the corresponding author.

## Abbreviations

The following abbreviations are used in this manuscript:

ASR	Automatic Speech Recognition
BIC	Bayesian Information Criterion
DNN	Deep Neural Network
DOA	Direction of Arrival
FA	False Alarm
GCC	Generalized Cross-correlation
GCC-PHAT	Generalized Cross-correlation with Phase Transform
GMM-UBM	Gaussian Mixture Model-based Universal Background Model
GNN	Graph Neural Networks
HDC	Hybrid Diarization Clustering
JFA	Joint Factor Analysis
MDM	Multiple Distant Microphones
MFCC	Mel Frequency Cepstral Coefficients
PHAT	Phase Transform
SAD	Speech Activity Detection
SC	Spectral Clustering
SE	Squeeze-and-Excitation
SMF	Similarity Matrix Fusion
TDNN	Time Delay Neural Network
TDOA	Time Difference of Arrival

## References

[1] Chen S., Gopalakrishnan P. (1998). Speaker, environment and channel change detection and clustering via the Bayesian Information Criterion. Proc. DARPA Broadcast News Transcription and Understanding Workshop.

[2] Tritschler A., Gopinath R.A. Improved speaker segmentation and segments clustering using the bayesian information criterion. Proceedings of the Sixth European Conference on Speech Communication and Technology.

[3] Reynolds D.A., Quatieri T.F., Dunn R.B. (2000). Speaker verification using adapted gaussian mixture models. Digit. Signal Process..

[4] Kenny P., Reynolds D., Castaldo F. (2010). Diarization of telephone conversations using factor analysis. IEEE J. Sel. Top. Signal Process..

[5] Dehak N., Kenny P.J., Dehak R., Dumouchel P., Ouellet P. (2011). Front-end factor analysis for speaker verification. IEEE Trans. Audio Speech Lang. Process..

[6] Variani E., Lei X., McDermott E., Moreno I.L., Gonzalez-Dominguez J. Deep Neural Networks for small footprint text-dependent speaker verification. Proceedings of the 2014 IEEE International Conference on Acoustics, Speech and Signal Processing (ICASSP).

[7] Heigold G., Moreno I., Bengio S., Shazeer N. End-to-end text-dependent speaker verification. Proceedings of the 2016 IEEE International Conference on Acoustics, Speech and Signal Processing (ICASSP).

[8] Wang Q., Downey C., Wan L., Mansfield P.A., Moreno I.L. Speaker diarization with LSTM. Proceedings of the 2018 IEEE International Conference on Acoustics, Speech and Signal Processing (ICASSP).

[9] Wang J., Xiao X., Wu J., Ramamurthy R., Rudzicz F., Brudno M. Speaker diarization with session-level speaker embedding refinement using graph neural networks. Proceedings of the ICASSP 2020—2020 IEEE International Conference on Acoustics, Speech and Signal Processing (ICASSP).

[10] Singh P., Kaul A., Ganapathy S. Supervised hierarchical clustering using graph neural networks for speaker diarization. Proceedings of the ICASSP 2023—2023 IEEE International Conference on Acoustics, Speech and Signal Processing (ICASSP).

[11] Xia W., Lu H., Wang Q., Tripathi A., Huang Y., Moreno I.L., Sak H. Turn-to-diarize: Online speaker diarization constrained by transformer transducer speaker turn detection. Proceedings of the ICASSP 2022—2022 IEEE International Conference on Acoustics, Speech and Signal Processing (ICASSP).

[12] Jeoung Y.-R., Yang J.-Y., Choi J.-H., Chang J.-H. Improving transformer-based end-to-end speaker diarization by assigning auxiliary losses to attention heads. Proceedings of the ICASSP 2023—2023 IEEE International Conference on Acoustics, Speech and Signal Processing (ICASSP).

[13] Kanda N., Xiao X., Gaur Y., Wang X., Meng Z., Chen Z., Yoshioka T. Transcribe-to-diarize: Neural speaker diarization for unlimited number of speakers using end-to-end speaker-attributed ASR. Proceedings of the ICASSP 2022—2022 IEEE International Conference on Acoustics, Speech and Signal Processing (ICASSP).

[14] Fang X., Ling Z.H., Sun L., Niu S.T., Du J., Liu C., Sheng Z.C. A deep analysis of speech separation guided diarization under realistic conditions. Proceedings of the 2021 Asia-Pacific Signal and Information Processing Association Annual Summit and Conference (APSIPA ASC).

[15] Morrone G., Cornell S., Raj D., Serafini L., Zovato E., Brutti A., Squartini S. Low-latency speech separation guided diarization for telephone conversations. Proceedings of the 2022 IEEE Spoken Language Technology Workshop (SLT).

[16] He M.-K., Du J., Liu Q.-F., Lee C.-H. (2023). ANSD-ma-MSE: Adaptive Neural speaker diarization using memory-aware multi-speaker embedding. IEEE/ACM Trans. Audio Speech Lang. Process..

[17] Yang G., He M., Niu S., Wang R., Yue Y., Qian S., Wu S., Du J., Lee C.-H. Neural speaker diarization using memory-aware multi-speaker embedding with sequence-to-sequence architecture. Proceedings of the ICASSP 2024—2024 IEEE International Conference on Acoustics, Speech and Signal Processing (ICASSP).

[18] Gebru I.D., Ba S., Li X., Horaud R. (2018). Audio-Visual speaker diarization based on spatiotemporal Bayesian fusion. IEEE Trans. Pattern Anal. Mach. Intell..

[19] Fujita Y., Kanda N., Horiguchi S., Xue Y., Nagamatsu K., Watanabe S. End-to-end neural speaker diarization with self-attention. Proceedings of the 2019 IEEE Automatic Speech Recognition and Understanding Workshop (ASRU).

[20] Fujita Y., Kanda N., Horiguchi S., Nagamatsu K., Watanabe S. (2019). End-to-end neural speaker diarization with permutation-free objectives. arXiv.

[21] Bost X., Linares G., Gueye S. Audiovisual speaker diarization of TV series. Proceedings of the 2015 IEEE International Conference on Acoustics, Speech and Signal Processing (ICASSP).

[22] Xu E.Z., Song Z., Tsutsui S., Feng C., Ye M., Shou M.Z. Ava-AVD: Audio-visual speaker diarization in the wild. Proceedings of the 30th ACM International Conference on Multimedia 2022.

[23] Vryzas N., Vrysis L., Dimoulas C. (2021). Audiovisual speaker indexing for web-TV automations. Expert Syst. Appl..

[24] Ellis D.P.W., Liu J.C. Speaker turn segmentation based on between-channel differences. Proceedings of the NIST Meeting Recognition Workshop at ICASSP 2004.

[25] Anguera X., Wooters C., Hernando J. (2007). Acoustic beamforming for speaker diarization of meetings. IEEE Trans. Audio Speech Lang. Process..

[26] Vijayasenan D., Valente F., Bourlard H. (2011). An information theoretic combination of MFCC and TDOA features for speaker diarization. IEEE Trans. Audio Speech Lang. Process..

[27] Araki S., Fujimoto M., Ishizuka K., Sawada H., Makino S. A DOA based speaker diarization system for real meetings. Proceedings of the 2008 Hands-Free Speech Communication and Microphone Arrays.

[28] Koh E.C., Sun H., Nwe T.L., Nguyen T.H., Ma B., Chng E.S., Li H., Rahardja S. Using direction of arrival estimate and acoustic feature information in speaker diarization. Proceedings of the Interspeech 2007, 8th Annual Conference of the International Speech Communication Association.

[29] Zheng S., Huang W., Wang X., Suo H., Feng J., Yan Z. A real-time speaker diarization system based on spatial spectrum. Proceedings of the ICASSP 2021—2021 IEEE International Conference on Acoustics, Speech and Signal Processing (ICASSP).

[30] Horiguchi S., Takashima Y., Garcia P., Watanabe S., Kawaguchi Y. Multi-channel end-to-end neural diarization with distributed microphones. Proceedings of the ICASSP 2022—2022 IEEE International Conference on Acoustics, Speech and Signal Processing (ICASSP).

[31] Xylogiannis P., Vryzas N., Bountourakis V., Dimoulas C. Multichannel speaker diarization with arbitrary microphone arrays. Proceedings of the AES Europe 2023: 154th Audio Engineering Society Convention (AES Europe 2023: 154th Audio Engineering Society Convention); Audio Engineering Society.

[32] Tsipas N., Vrysis L., Konstantoudakis K., Dimoulas C. (2020). Semi-supervised audio-driven TV-news speaker diarization using Deep Neural Embeddings. J. Acoust. Soc. Am..

[33] Snyder D., Garcia-Romero D., Sell G., Povey D., Khudanpur S. X-vectors: Robust DNN embeddings for speaker recognition. Proceedings of the 2018 IEEE International Conference on Acoustics, Speech and Signal Processing (ICASSP).

[34] Desplanques B., Thienpondt J., Demuynck K. ECAPA-TDNN: Emphasized channel attention, propagation and aggregation in TDNN based speaker verification. Proceedings of the Interspeech, 2020.

[35] Knapp, Carter G. (1976). The generalized correlation method for estimation of time delay. IEEE Trans. Acoust. Speech Signal Process..

[36] Anguera X., Wooters C., Pardo J.M. (2006). Robust speaker diarization for meetings: ICSI RT06S meetings evaluation system. Machine Learning for Multimodal Interaction.

[37] Silero Team (2021). Silero VAD: Pre-trained enterprise-grade Voice Activity Detector (VAD), Number Detector and Language Classifier. Retrieved March. https://github.com/snakers4/silero-vad.

[38] Ning H., Liu M., Tang H., Huang T.S. A spectral clustering approach to speaker diarization. Proceedings of the Interspeech, 2006.

[39] Park T.J., Han K.J., Kumar M., Narayanan S. (2020). Auto-tuning spectral clustering for speaker diarization using normalized maximum eigengap. IEEE Signal Process. Lett..

[40] Vryzas N., Tsipas N., Dimoulas C. (2020). Web radio automation for audio stream management in the era of Big Data. Information.

[41] Park T.J., Kanda N., Dimitriadis D., Han K.J., Watanabe S., Narayanan S. (2022). A review of speaker diarization: Recent advances with deep learning. Comput. Speech Amp Lang..

